# Heat Risk Perceptions and Coping Strategies of the Unhoused

**DOI:** 10.3390/ijerph21060737

**Published:** 2024-06-05

**Authors:** Brett W. Robertson, Kirstin Dow, Julie Salinas, Susan L. Cutter

**Affiliations:** 1 College of Information and Communications, University of South Carolina, Columbia, SC 29208, USA; 2 Department of Geography, University of South Carolina, Columbia, SC 29208, USA; kdow@sc.edu (K.D.); scutter@mailbox.sc.edu (S.L.C.); 3 Department of Educational Psychology & Research, University of South Carolina, Columbia, SC 29208, USA; salinaj@email.sc.edu

**Keywords:** excessive heat, extreme heat, unhoused populations, homelessness, theory of planned behavior

## Abstract

The escalating awareness of heat-related risks and the associated imperative to enhance preparedness strategies at various levels has spurred a growing emphasis on disseminating knowledge about heat vulnerability. These efforts aim to equip diverse stakeholders with practical heat planning and forecasting tools. The success of these communication initiatives hinges on understanding the nuanced perceptions of risk and the priority assigned to addressing heat as a health risk. This paper delves explicitly into the unhoused population’s risk perceptions and coping strategies. Acknowledged as one of the most underserved and vulnerable groups to extreme heat, unhoused individuals face prolonged exposure, a heightened frequency of mental and physical health issues, and limited coping resources during extreme heat events. Despite widespread acknowledgment of their vulnerability, little attention has been directed towards researching health behavior within this demographic about excessive heat. We developed a survey instrument using the theory of planned behavior (TPB) to bridge this gap. We collected quantitative survey data from unhoused populations in Columbia, South Carolina, an area of the United States that experiences extreme heat events and has a sizeable unhoused population. Using a series of hierarchical multiple regression models, our findings indicate that TPB variables predict the intention to avoid the heat while considering additional coping strategies common among unhoused individuals. These findings offer valuable insights for public health researchers, practitioners, and community officials engaged in direct interactions with unhoused populations, informing how this underserved group manages excessive heat exposure.

## 1. Introduction

Rising awareness of heat risks by the public and local governments reveals the critical need to enhance extreme heat preparedness strategies across all scales, from individuals to households, and across various jurisdictions, including local governments, states, and federal agencies. With the increasing frequency and intensity of heatwaves due to climate change, it is imperative to prioritize the effective communication of heat vulnerability knowledge to individuals and develop practical tools for heat planning and forecasting. These measures are now on the agenda for many communities aiming to mitigate the impacts of extreme heat [1,2]. The overall effectiveness of these efforts is influenced by community members’ perceptions of risk and their coping strategies, which can vary significantly, as shown in national surveys [3,4].

Using a quantitative survey, this paper focuses on one element of this multi-faceted issue: unhoused people’s risk perceptions and coping strategies during times of extreme heat. Unhoused populations are among the most vulnerable to extreme heat due to prolonged exposure, a higher incidence of mental and physical health issues, and limited coping resources [5,6]. Moreover, the vulnerabilities of unhoused populations extend beyond extreme heat, encompassing a range of environmental changes and climate shocks, such as flooding, cold waves, and air pollution, which exacerbate their precarious living conditions and health risks [7,8,9]. These vulnerabilities highlight the need for an adaptive capacity beyond the household level to effectively manage climate risks.

Despite their heightened vulnerabilities, unhoused populations are rarely the focus of risk perception literature. In Maricopa County, Arizona, for example, deaths among the unhoused accounted for 45% (290 individuals) of all heat-associated deaths in 2022 [10]. While this vulnerability is broadly acknowledged, research on health-seeking behaviors and coping strategies among the unhoused during extreme heat events remains limited [11,12]. Addressing this gap is crucial for developing targeted interventions that can save lives and improve the overall well-being of unhoused individuals facing multiple environmental hazards [13,14].

To address this gap, we developed a survey questionnaire grounded in the theory of planned behavior (TPB), augmented with additional questions on coping strategies, including the perceived value of heat-related information and comprehension difficulty. TPB, a well-established theoretical model, helps identify attitudes, subjective norms, and behaviors that underlie the intention to avoid heat exposure. This study aims to enhance understanding of how unhoused individuals perceive the severity of heat risks and their strategies to avoid heat-related illnesses.

### Theory of Planned Behavior

The theory of planned behavior (TPB) has been widely used across disciplines to understand the mechanisms of conscious decision-making, including decisions related to health behaviors and environmental hazards [15,16]. TPB is particularly useful for this study because it provides a structured framework to analyze how unhoused individuals form intentions to avoid heat exposure. By understanding their attitudes towards heat, the social pressures they face (subjective norms), and their perceived control over avoiding heat, we can gain insights into their decision-making processes and identify potential barriers to effective coping strategies. This framework allows us to dissect the complex interplay of beliefs and behaviors that influence how unhoused individuals perceive and respond to extreme heat risks [17].

The primary constructs of TPB include attitudes, subjective norms, perceived behavioral control (PBC), and intention. Attitudes reflect the sum of value-weighted beliefs that performing a specific behavior will result in a desired outcome. Subjective norms represent the influence of social pressure, combining the perceived expectations of others regarding the behavior. Perceived behavioral control acknowledges that not all behaviors are under an individual’s direct volitional control [18]. These variables are crucial because they help identify the specific beliefs and perceptions that drive behavior, allowing us to tailor interventions more effectively.

Applying TPB to this study is particularly relevant because the unhoused population faces unique challenges that influence their health behaviors and coping strategies. The limited use of TPB in investigating the health behaviors of unhoused people includes research on the intent to be screened for HIV [19], rule-following behavior among youths in shelters [20], the uptake of outreach programs to support recovery [21], the impact of housing on medication adherence [22], and the likelihood of seeking long-term addiction treatment [23]. These studies demonstrate TPB’s utility in understanding behavior in vulnerable populations, but also highlight that the significance of TPB constructs can vary among different contexts and populations [21].

For this study, using TPB helps to identify the attitudes, norms, and perceived controls specific to the unhoused population in the context of extreme heat. This is important because it allows for a nuanced understanding of how these individuals perceive heat risks and what influences their coping strategies. However, TPB also has limitations. For example, it may not fully capture the complexity of risk perceptions and adaptive behaviors in extreme heat scenarios. Scholars such as Shi and Kim (2019) and Ng (2022) [24,25] suggest incorporating additional risk perception variables to enhance TPB’s predictive ability regarding behavioral intentions. Rimal and Real (2003) [26] emphasize that many risk perception models struggle to distinguish cause from effect, highlighting the need to carefully consider perceived risk variables to account for varying demographic and psychological characteristics. Additionally, social policies and climate-responsive social protection measures are critical for supporting vulnerable populations in the face of climate emergencies [27,28].

By addressing these limitations and leveraging the strengths of TPB, our study aims to provide valuable insights into how unhoused individuals perceive and respond to extreme heat. Therefore, we propose the following research questions to guide our inquiry:

RQ1: To what extent do the variables of TPB (attitudes about heat, subjective norms, and perceived behavioral control) predict the intention to avoid heat?

RQ2: To what extent do TPB’s variables (attitudes about heat, subjective norms, and perceived behavioral control) predict the intention to avoid heat when considering coping strategies?

## 2. Methods

In August and September 2022, we recruited individuals to participate in a survey about heat risk perceptions around the public library in downtown Columbia, SC, USA (Metropolitan Statistical Area population 837,092). This location was chosen because of the unhoused population that frequents this area, and because this region experiences several extreme heat periods in the summer months. The city of Columbia, SC, USA, experiences high summer temperatures and heat index values due to its humid subtropical climate, which includes long, hot, and humid summers.

This region also has a sizable unhoused community, with an 18 percent increase in homelessness reported in 2021, and currently 1500 individuals who are considered unhoused [29]. While the city provides some emergency and temporary housing for unhoused individuals and children, the services for homelessness in Columbia, SC, USA, are lacking due to insufficient resources, such as a limited number of clinicians to respond to incidents and inadequate funding to meet the growing needs of the unhoused population [30].

Eligible participants were at least 18 years old and living in Columbia, SC, USA. A total of 58 individuals participated, with 69% (*n* = 40) considering themselves unhoused. We included 18 individuals who considered themselves housed in the final dataset, as some of these individuals indicated they lived in temporary transitional housing, while others lived with family or friends. Given that the experience of living outside during extreme heat conditions is very different from someone who has access to shelter, even temporarily [31], it is useful to contrast these groups. While this sample size may be low, researchers (e.g., [32]) continue to stress that negotiating access to underserved populations is incredibly difficult in social science research, and lower sample sizes may be acceptable when collecting data from a vulnerable population.

The mean age of all participants was fifty years (*SD* = 13.49). The sample included forty-eight male and ten female participants. Of these participants, twenty were White, thirty-five were Black or African American, one was Hispanic or Latino, one was Native Hawaiian or Other Pacific Islander, and one identified as Other. Table 1 contains a full breakdown of demographic characteristics between the housed and unhoused groups.

The Institutional Review Board (IRB) approved this study at the authors’ institution. The survey questions were adapted from those created by the Eastern Research Group (2018) [33], in partnership with the National Weather Service, which used the TPB as part of a more extensive national weather messaging study, to allow for comparisons where appropriate. We also developed questions in our survey to understand heat health risk’s social and cultural components. Those interested in participating were first asked to read an informed consent document. After reading the document, participants gave informed consent by a verbal “yes” to the researcher. Researchers then began the survey and guided each participant through the questions. The survey took approximately 20 min to complete, and participants were given a USD 10 gift card once finished. See Appendix A for full survey instrument.

Composite scores were created by calculating the average response score for each participant for a construct. Intention to avoid the heat was assessed using a single-item measure. This item evaluated the degree to which the participant intended to avoid the heat (*M* = 6.40, *SD* = 0.88). Subjective norms towards groups’ beliefs on excessive heat information were assessed using a three-item measure. Participants rated the degree to which they felt that (a) their friends, (b) those important to them, and (c) their family expected them to know something about excessive heat. These items were combined to create a scale with α = 0.91, *M* = 5.46, and *SD* = 1.67. Attitude towards the actions needed to avoid the heat was assessed using a seven-point semantic scale. Participants rated the degree to which they would describe the actions needed to avoid the heat from (a) difficult to easy and (b) unpleasant to pleasant. These two items were combined to create a scale with α = 0.60, *M* = 3.57, and *SD* = 2.0. Perceived behavioral control of avoiding the heat was assessed using a single-item measure. Participants rated the degree to which they believed avoiding the heat was up to themselves (*M* = 6.25 and *SD* = 1.11).

In addition to the TPB variables, we included five sets of variables that provide additional insight into the TBP responses. They are perceived susceptibility and severity of extreme heat exposure, practice of adaptive behaviors, perceived value of heat information, and difficulty comprehending available heat information.

Perceived susceptibility to exposure to excessive heat was assessed using a two-item measure. Participants rated the degree to which they felt (a) themselves and (b) their community were likely to be exposed to excessive heat. These items were combined to create a scale with α = 0.76, *M* = 5.93, and *SD* = 1.35.

Perceptions of the severity of being affected by excessive heat were assessed using a two-item measure. Participants rated the degree to which they felt how seriously (a) themselves and (b) their local community would be affected by excessive heat. These items were combined to create a scale α = 0.77, *M* = 6.0, and *SD* = 1.65.

Adaptive behaviors towards excessive heat were assessed using a ten-item measure. Participants rated the degree to which (a) they used air conditioning, (b) refrained from using air conditioning because it would increase their power bill, (c) their air conditioning cooled their whole residence, (d) they were aware of resources offered by their community, (e) they avoided being outside, (f) they stayed inside, (g) they made efforts to stay hydrated, (h) the heat influenced them to change their schedule, (i) they visited family or friends, and (j) they visited public places that had air conditioning during excessive heat. These items were combined to create a scale with α = 0.72, *M* = 4.93, and *SD* = 0.99.

The perceived value of heat information was assessed using a four-item measure. Participants rated the degree to which they felt that understanding the risks imposed by excessive heat was (a) important, (b) useful, (c) valuable, and (d) beneficial. These items were combined to create a scale with α = 0.70, *M* = 6.65, and *SD* = 0.62.

The difficulty of comprehending heat information was assessed using a four-item measure. Participants rated the degree to which they felt (a) they could not make sense of excessive heat information, (b) they did not know how to separate fact from fiction, (c) excessive heat information was too technical for them to understand, and (d) they did not understand excessive heat information even if they tried. These items were combined to create a scale with α = 0.78, *M* = 2.67, and *SD* = 1.38.

## 3. Results

To understand some key differences between the housed and unhoused groups as background for our study, an independent samples *t*-test was conducted for each variable in the survey to find any significant difference in variable responses. Overall, the housed and unhoused respondents showed a strong intention to avoid the heat (Table 2). The unhoused group had lower means for adaptive behaviors, attitudes, and perceived value of information than the housed group. However, *t*-tests comparing the composite means found only the difference in the severity of impacts to be significant (*t* = −2.413; *p* = 0.025). Specific variable differences are further discussed using independent samples *t*-test in Table 2.

A hierarchical multiple regression was used to answer RQ1, which addressed the TPB variables’ influence on intention to avoid the heat, with separate regressions run for the unhoused and housed participants. The first model contained subjective norms, attitudes, and perceived behavioral control in the first block. The second block contained susceptibility, severity, adaptive behaviors, value of information, and comprehension difficulty constructs and was entered to create the second model. Intention remained as the dependent variable for all regression analyses. Note that variations in sample size appear in all regression models, as participants may not have answered every question.

Results for Model 1 for the unhoused group showed that the TPB predictor variables accounted for 17.9% of the variance in intention to avoid the heat (R^2^ adj = 0.179, *F* (3, 33) = 3.62, and *p* = 0.023). Only one of the predictor variables had a statistically significant effect on the intention to avoid the heat: perceived control (β = 0.470 and *p* = 0.005). Model 1 was not significant for the housed group. The results for RQ2 are presented in Table 3.

To investigate RQ2, which focused on the influence of TPB variables on intention to avoid the heat while taking into account coping strategies, we added five additional predictor variables (susceptibility, severity, adaptive behaviors, value of information, and comprehension difficulty). Again, hierarchical multiple regression was used and separate regressions were run for the unhoused and housed participants.

For this second enhanced TPB model, the five additional predictor variables were entered into the model as the second block, following the first block with the original TPB constructs. The results for the unhoused group showed that the second model accounted for 38.5% of the variance in intention (R^2^ adj = 0.385, *F* (8, 28) = 3.81, and *p* = 0.004). Adding these five additional variables in the second block increased the R^2^ of ΔR^2^ to 0.274 (*F* (5, 28) = 3.20 and *p* = 0.021). These additional variables accounted for 27.4% of the variance in intention beyond the variance accounted for by the TPB variables. The four predictor variables that had a significant effect on the intention to avoid heat are as follows: subjective norms (β = −0.383 and *p* = 0.016); perceived control (β = 0.583 and *p* < 0.001); value of information (β = 0.384 and *p* = 0.025); and information comprehension (β = −0.433 and *p* = 0.006).

Although Model 2 is not significant for the housed group, R^2^ increased by ΔR^2^ = 0.596 (*F* (5, 8) = 4.08 and *p* = 0.039). These results are presented in Table 4.

## 4. Discussion

This study examined the differences in perception of excessive heat risks and coping strategies between unhoused and housed populations in Columbia, South Carolina, USA. Our findings underscored important differences in the adaptive strategies employed by these two groups, shedding light on the complex intersectionality of homelessness, climate-related challenges, and communication for this population.

The central finding of our study is the significant difference in perceived severity of heat impacts between unhoused and housed populations. Unhoused participants reported a higher perceived severity of excessive heat, which aligns with their heightened vulnerability due to living conditions and limited access to resources. This underscores the necessity for targeted interventions for unhoused individuals, emphasizing the heightened risks and promoting awareness campaigns tailored to their unique circumstances.

In comparing our variables using the TPB, we noted that the severity of impacts was the only variable that statistically differed between the two groups. Severity is one of the factors influencing an individual’s intention to engage in a specific behavior [24,25]. Including severity in the TPB recognizes that an individual’s perception of the seriousness or gravity of a particular behavior can impact their intention to perform or avoid that behavior, which, in the case of this study, involves avoiding the heat. Severity involves an individual’s assessment of the potential harm or negative consequences associated with a specific health-related action or inaction. The unhoused population in our study had a higher perceived severity level than our housed participants. This suggests that the unhoused participants in our study understood that excessive heat is serious. The heightened perception of severity among unhoused populations may be rooted in their unique living conditions, increased vulnerability, and limited access to resources that can mitigate the impact of excessive heat [8]. Decision-makers and practitioners should leverage this insight to develop interventions that communicate the serious consequences of excessive heat to unhoused individuals, potentially enhancing their intention to adopt protective behaviors.

Using a series of regression models to compare behaviors for our unhoused and housed participants, the model using the TPB predictors alone was significant for our unhoused population, accounting for 17.9% of the variances in the intention to avoid the heat. Perceived behavior control was a significant predictor in this model. This finding suggests that an individual’s belief in their ability to control or manage their behavior in response to heat is a key factor influencing their intention to avoid it. The significance of perceived behavioral control implies that, despite the challenges associated with homelessness, individuals may harbor a sense of agency and confidence in their capacity to navigate and manage responses to excessive heat [34]. This finding aligns with TPB’s emphasis on performing a behavior’s perceived ease or difficulty. Meanwhile, integral components of the theory, attitudes toward the heat, and subjective norms did not emerge as significant predictors in this specific context, suggesting that the practical aspects of control over behavior could hold greater sway in shaping intentions to avoid extreme heat among the unhoused. Recognizing the prominence of perceived behavioral control provides valuable insights for tailoring interventions and support systems that enhance individuals’ confidence and agency in addressing the challenges posed by excessive heat within the unhoused community [35].

Expanding upon TPB to identify additional factors that may influence the intention to avoid the heat, our enhanced model, which added susceptibility, severity, adaptive behaviors, value of information, and comprehension difficulty to the TPB variables (subjective norms, attitude, and perceived behavioral control), was only significant for the unhoused population. The enhanced model accounted for 38.5% of the variance in intention, with subjective norms, perceived control, the value of information, and comprehension difficulty being significant predictors. The significance of subjective norms, which was only significant in this enhanced model, implies that the social influences and perceived expectations from others within the unhoused community play a crucial role in shaping intentions related to avoiding extreme heat [36]. Perhaps communal dynamics and shared beliefs influence individual decisions regarding heat mitigation strategies [37]. It is noted that unhoused individuals often rely on other peers who are unhoused for important information [38], and understanding how social capital is navigated in times of extreme weather events within the unhoused community warrants more attention.

Furthermore, the value of information emerged as a significant predictor, indicating that the unhoused population places importance on information relevant to heat avoidance. This underscores the potential effectiveness of targeted health communication campaigns in empowering individuals to make informed decisions about protective behaviors in extreme heat [39]. Notably, the comprehension difficulty was also significant, suggesting that interventions need to be tailored to ensure that information is presented in a clear and accessible manner. This highlights the importance of considering the literacy levels and communication barriers within the unhoused population when designing health communication initiatives [40].

The study’s findings have practical implications for tailored interventions addressing excessive heat in unhoused and housed populations. Since severity is a distinguishing factor between our housed and unhoused participants, interventions for the unhoused should prioritize awareness campaigns highlighting the heightened risks associated with extreme heat [41]. For the housed populations, emphasizing perceived behavioral control in promoting heat-avoidance behaviors is crucial. Our enhanced model’s significance for the unhoused underscores the importance of thoughtful interventions, including community-driven efforts and support systems. Practical application suggestions include developing targeted health communication programs for the unhoused, integrating social influences, perceived control, and clear communication strategies. Overall, these findings guide the development of context-specific initiatives derived from TPB that address the distinct needs of both populations, promoting effective heat mitigation and enhancing overall community resilience in the face of extreme heat events [37]. Our findings contribute to the broader literature on climate mitigation, vulnerability, and risk communication by providing empirical evidence on the specific needs of unhoused populations in the face of extreme heat. Policymakers and practitioners should consider these insights when designing and implementing heat mitigation strategies, ensuring that they are tailored to address the distinct vulnerabilities of unhoused individuals.

Despite the insights gained from our study on excessive heat among unhoused and housed populations, several limitations should be considered. First, the potential for sampling bias may limit the generalizability of findings, as participants were recruited from specific locations in the specific region of the authors, potentially overlooking the diversity within these populations. While some may consider our sample small, recruiting unhoused populations to participate in academic research is a noted challenge—and generally, smaller samples are seen as acceptable [32]. The reliance on self-reported data introduces the possibility of social desirability bias, where participants may provide responses influenced by perceived social norms. The study primarily relies on self-reported intentions as a single-item measure, potentially overlooking discrepancies between stated intentions and actual behaviors. Furthermore, this work did not comprehensively address the dynamic nature of homelessness and the potential impact of external factors. Future research should address these limitations to enhance the robustness and applicability of findings to other underserved populations.

This work serves as an exploratory analysis to better understand how unhoused and housed populations—who may have access to some form of shelter—understand their risk of excessive heat. Unhoused populations face additional vulnerabilities in hazard events like extreme heat events, and our work situated some of the unique predictors that allow us to understand risk perceptions among this underserved group. Because homelessness in the United States is on the rise [42], and extreme heat events are going to continue, we must consider how underserved populations, like the unhoused, cope with the heat in order to better serve their needs in the future.

## Figures and Tables

**Table 1:** Demographic characteristics of housed and unhoused persons in Columbia, SC, USA.

Characteristic	Housed *n* = 18 (%)	Unhoused *n* = 40 (%)
Age in years: mean (SD)		
18–50 years	9 (50.0%)	15 (37.5%)
51 or more years	9 (50.0%)	25 (62.5%)
Gender		
Male	15 (83.3%)	33 (82.5%)
Female	3 (16.7)	7 (17.5%)
Race and Ethnicity		
White	2 (11.1%)	18 (45.0%)
Black or African American	15 (83.5%)	20 (50.0%)
Hispanic or Latino	-	1 (2.5%)
Asian	-	-
American Indian or Alaskan Native	-	-
Native Hawaiian or Other Pacific Islander	1 (5.6%)	-
Other	-	1 (2.5%)
Education Level		
No schooling	-	1 (2.5%)
Some schooling completed	3 (16.7%)	6 (15.0%)
High school/general education diploma	10 (55.6%)	23 (57.5%)
Some college/associates/vocational degree	3 (16.7%)	9 (22.5%)
College graduate/bachelor’s degree	2 (11.1%)	-
Advanced degree	-	1 (2.5%)
Income		
Less than USD 14,580	7 (50.0%)	32 (88.9%)
USD 14,581–30,000	4 (28.6%)	3 (8.3%)
USD 30,001–60,000	1 (7.1%)	1 (2.8%)
USD 60,001–90,000	1 (7.1%)	-
USD 90,001–120,000	1 (7.1%)	-
USD 120,001 or more	-	-

**Table 2:** Independent *t*-tests for housed and unhoused group composite variables included in hierarchical regression models (variation in Ns are masked).

Variable	Housed (*n* = 17)		Unhoused (*n* = 37)	
	*M*	*SD*	*M*	*SD*
Intention	6.18	1.29	6.59	0.50
Subjective Norms	5.35	1.96	5.57	1.58
Attitude	4.18	2.21	3.39	1.92
Perceived Behavior Control (PBC)	5.65	1.66	6.49	0.65
Susceptibility	5.74	1.47	6.03	1.27
Severity	5.06	2.12	6.49	1.18
Adaptive Behaviors	5.33	1.23	4.86	0.74
Value of Information	6.74	0.51	6.59	0.68
Comprehension Difficulty	2.49	1.20	2.82	1.49

Note. *M* = mean. *SD* = standard deviation.

**Table 3:** Hierarchical regression results for housed and unhoused groups using TPB variables.

Sample Group	Predictor	Model R^2^	Adj R^2^	ΔR^2^	β	*p*
Housed		0.170	−0.022	0.170		
	Subjective Norms				0.206	0.448
	Attitude				0.300	0.272
	PBC				−0.059	0.819
Unhoused		0.248 *	0.179 *	0.248 *		
	Subjective Norms				−0.211	0.194
	Attitude				0.034	0.834
	PBC				0.470	0.005

Note. * *p* < 0.05. β = standardized multiple regression coefficient (beta weight).

**Table 4:** Hierarchical regression results for housed and unhoused groups using TPB and additional coping strategies variables.

Sample Group	Predictor	Model R^2^	Adj R^2^	ΔR^2^	β	*p*
Housed (n = 17)		0.766	0.532	0.596		
	Subjective Norms				−0.938	0.055
	Attitude				0.594	0.026
	PBC				−0.205	0.292
	Susceptibility				−0.676	0.049
	Severity				0.473	0.128
	Adaptive Behaviors				10.07	0.007
	Perceived Value of Information				−0.030	0.908
	Comprehension Difficulty				−0.249	0.263
Unhoused (n = 37)		0.521 *	0.385 *	0.274 *		
	Subjective Norms				−0.383	0.016
	Attitude				−0.108	0.497
	PBC				0.583	<0.001
	Susceptibility				0.072	0.725
	Severity				−0.183	0.339
	Adaptive Behaviors				−0.025	0.862
	Perceived Value of Information				0.384	0.025
	Information Comprehension				−0.433	0.006

Note. * *p* < 0.05. β = Standardized multiple regression coefficient (beta weight).

## Data Availability

The data presented in this study are available on reasonable request from the corresponding author.

## Appendix A. Survey Items

### Appendix A.1. Survey Questionnaire

Instructions

Now, we have a few questions about how you think about excessive heat and how you experience it. “Excessive heat” is a combination of high temperatures and high humidity. Excessive heat prevents your body from keeping its normal temperature and may result in heat illness such as heat cramps, or fainting. Using this information, please answer the following questions to the best of your knowledge.

Using a scale from 1 to 7, where 1 means not likely at all and 7 means extremely likely, how likely are you to be exposed to excessive heat?

[1] Extremely Unlikely
[2] Very Unlikely
[3] Unlikely
[4] Neutral
[5] Likely
[6] Very Likely
[7] Extremely Likely

Using a scale from 1 to 7, where 1 means not likely at all and 7 means extremely likely, how likely are people in your local community to be exposed to excessive heat?

[1] Extremely Unlikely
[2] Very Unlikely
[3] Unlikely
[4] Neutral
[5] Likely
[6] Very Likely
[7] Extremely Likely

Using a scale from 1 to 7, where 1 means not serious at all and 7 means extremely serious, how seriously would you be affected by excessive heat?

[1] Not serious at all
[2] -
[3] -
[4] -
[5] -
[6] -
[7] Extremely Serious

Using a scale from 1 to 7, where 1 means not serious at all and 7 means extremely serious, how seriously would people in your local community be affected by excessive heat?

[1] Not serious at all
[2] -
[3] -
[4] -
[5] -
[6] -
[7] Extremely Serious

Using a scale from 1 to 7, where 1 means strongly disagree and 7 means strongly agree, how do you rate your agreement or disagreement with the following statements:

I use air-conditioning during excessive heat periods.

[1] Strongly disagree
[2] Disagree
[3] Somewhat disagree
[4] Neither agree nor disagree
[5] Somewhat agree
[6] Agree
[7] Strongly agree

I don’t use air-conditioning during excessive heat because it increases my power bill.

[1] Strongly disagree
[2] Disagree
[3] Somewhat disagree
[4] Neither agree nor disagree
[5] Somewhat agree
[6] Agree
[7] Strongly agree

My air-conditioning cools the whole residence adequately.

[1] Strongly disagree
[2] Disagree
[3] Somewhat disagree
[4] Neither agree nor disagree
[5] Somewhat agree
[6] Agree
[7] Strongly agree

I am aware of resources offered by my community for staying cool during excessive heat periods.

[1] Strongly disagree
[2] Disagree
[3] Somewhat disagree
[4] Neither agree nor disagree
[5] Somewhat agree
[6] Agree
[7] Strongly agree

I avoid being outside during excessive heat.

[1] Strongly disagree
[2] Disagree
[3] Somewhat disagree
[4] Neither agree nor disagree
[5] Somewhat agree
[6] Agree
[7] Strongly agree

I usually stay inside during excessive heat.

[1] Strongly disagree
[2] Disagree
[3] Somewhat disagree
[4] Neither agree nor disagree
[5] Somewhat agree
[6] Agree
[7] Strongly agree

I make an effort to stay hydrated during excessive heat.

[1] Strongly disagree
[2] Disagree
[3] Somewhat disagree
[4] Neither agree nor disagree
[5] Somewhat agree
[6] Agree
[7] Strongly agree

Excessive heat influences me to change my schedule.

[1] Strongly disagree
[2] Disagree
[3] Somewhat disagree
[4] Neither agree nor disagree
[5] Somewhat agree
[6] Agree
[7] Strongly agree

I visit family or friends to avoid excessive heat.

[1] Strongly disagree
[2] Disagree
[3] Somewhat disagree
[4] Neither agree nor disagree
[5] Somewhat agree
[6] Agree
[7] Strongly agree

I visit public places in my community that have air conditioning to avoid excessive heat.

[1] Strongly disagree
[2] Disagree
[3] Somewhat disagree
[4] Neither agree nor disagree
[5] Somewhat agree
[6] Agree
[7] Strongly agree

Using a scale from 1 to 7, where 1 means strongly disagree and 7 means strongly agree, how do you rate your agreement or disagreement with the following.

Understanding the risks posed by excessive heat is: Important

[1] Strongly disagree
[2] Disagree
[3] Somewhat disagree
[4] Neither agree nor disagree
[5] Somewhat agree
[6] Agree
[7] Strongly agree

Understanding the risks posed by excessive heat is: Useful

[1] Strongly disagree
[2] Disagree
[3] Somewhat disagree
[4] Neither agree nor disagree
[5] Somewhat agree
[6] Agree
[7] Strongly agree

Understanding the risks posed by excessive heat is: Valuable

[1] Strongly disagree
[2] Disagree
[3] Somewhat disagree
[4] Neither agree nor disagree
[5] Somewhat agree
[6] Agree
[7] Strongly agree

Understanding the risks posed by excessive heat is: Beneficial

[1] Strongly disagree
[2] Disagree
[3] Somewhat disagree
[4] Neither agree nor disagree
[5] Somewhat agree
[6] Agree
[7] Strongly agree

Using a scale from 1 to 7, where 1 means strongly disagree and 7 means strongly agree, how do you rate your agreement or disagreement with the following statements.

I can’t make sense of information about excessive heat.

[1] Strongly disagree
[2] Disagree
[3] Somewhat disagree
[4] Neither agree nor disagree
[5] Somewhat agree
[6] Agree
[7] Strongly agree

When it comes to information about excessive heat, I don’t know how to separate facts from fiction.

[1] Strongly disagree
[2] Disagree
[3] Somewhat disagree
[4] Neither agree nor disagree
[5] Somewhat agree
[6] Agree
[7] Strongly agree

Most information about excessive heat is too technical for me to understand.

[1] Strongly disagree
[2] Disagree
[3] Somewhat disagree
[4] Neither agree nor disagree
[5] Somewhat agree
[6] Agree
[7] Strongly agree

I don’t understand information about excessive heat even if I make an effort.

[1] Strongly disagree
[2] Disagree
[3] Somewhat disagree
[4] Neither agree nor disagree
[5] Somewhat agree
[6] Agree
[7] Strongly agree

Using a scale from 1 to 7, where 1 means strongly disagree and 7 means strongly agree, how do you rate your agreement or disagreement with the following statements.

My friends expect me to know something about excessive heat.

[1] Strongly disagree
[2] Disagree
[3] Somewhat disagree
[4] Neither agree nor disagree
[5] Somewhat agree
[6] Agree
[7] Strongly agree

Most people who are important to me expect me to know something about excessive heat.

[1] Strongly disagree
[2] Disagree
[3] Somewhat disagree
[4] Neither agree nor disagree
[5] Somewhat agree
[6] Agree
[7] Strongly agree

My family expects me to know something about excessive heat.

[1] Strongly disagree
[2] Disagree
[3] Somewhat disagree
[4] Neither agree nor disagree
[5] Somewhat agree
[6] Agree
[7] Strongly agree

Now we are going to ask about your attitudes toward excessive heat and how you may avoid it.

How would you describe the actions you need to take to avoid the heat? Where would you place it on this scale:

[1] Difficult
[2] -
[3] -
[4] -
[5] -
[6] -
[7] Easy

How would you describe the actions you need to take to avoid the heat? Where would you place it on this scale:

[1] Unpleasant
[2] -
[3] -
[4] -
[5] -
[6] -
[7] Pleasant

Using a scale from 1 to 7, where 1 means strongly disagree and 7 means strongly agree, how do you rate your agreement or disagreement with the following statements.

I am confident that I can avoid the heat.

[1] Strongly disagree
[2] Disagree
[3] Somewhat disagree
[4] Neither agree nor disagree
[5] Somewhat agree
[6] Agree
[7] Strongly agree

Avoiding the heat is up to me.

[1] Strongly disagree
[2] Disagree
[3] Somewhat disagree
[4] Neither agree nor disagree
[5] Somewhat agree
[6] Agree
[7] Strongly agree

In the past, I have taken actions to avoid heat.

[1] Strongly disagree
[2] Disagree
[3] Somewhat disagree
[4] Neither agree nor disagree
[5] Somewhat agree
[6] Agree
[7] Strongly agree

[Demographics Section]

Now, we’re going to conclude the survey with basic demographic questions.

Age (in years): ___________

Gender: _______________

What is the highest level of education you have completed?

[1] No schooling completed
[2] Some schooling completed
[3] High school graduate/GED
[4] Some college/associates/vocational degree
[5] College graduate/bachelor’s degree
[6] Advanced Degree

What best describes your race?

[1] White
[2] Black or African American
[3] Hispanic or Latino
[4] Asian
[5] American Indian or Alaska Native
[6] Native Hawaiian or Other Pacific Islander
[7] Other: [TEXT]

Thinking specifically about the past 12 months, what was your annual household income from all sources?

[1] Less than $14,580
[2] $14,581–30,000
[3] $30,001–60,000
[4] $60,001–90,000
[5] $90,001–120,000
[6] $120,001 and above
